# Thioflavin-positive tau aggregates complicating quantification of amyloid plaques in the brain of 5XFAD transgenic mouse model

**DOI:** 10.1038/s41598-021-81304-6

**Published:** 2021-01-15

**Authors:** Jisu Shin, Sohui Park, HeeYang Lee, YoungSoo Kim

**Affiliations:** Department of Pharmacy, Yonsei University, Incheon, 21983 Republic of Korea

**Keywords:** Fluorescence imaging, Alzheimer's disease

## Abstract

Transgenic mouse models recapitulating Alzheimer’s disease (AD) pathology are pivotal in molecular studies and drug evaluation. In transgenic models selectively expressing amyloid-β (Aβ), thioflavin S (ThS), a fluorescent dye with β-sheet binding properties, is widely employed to observe amyloid plaque accumulation. In this study, we investigated the possibility that a commonly used Aβ-expressing AD model mouse, 5XFAD, generates ThS-positive aggregates of β-sheet structures in addition to Aβ fibrils. To test this hypothesis, brain sections of male and female 5XFAD mice were double-stained with ThS and monoclonal antibodies against Aβ, tau, or α-synuclein, all of which aggregates are detected by ThS. Our results revealed that, in addition to amyloid plaques, 5XFAD mice express ThS-positive phospho-tau (p-tau) aggregates. Upon administration of a small molecule that exclusively disaggregates Aβ to 5XFAD mice for six weeks, we found that the reduction level of plaques was smaller in brain sections stained by ThS compared to an anti-Aβ antibody. Our findings implicate that the use of ThS complicates the quantification of amyloid plaques and the assessment of Aβ-targeting drugs in 5XFAD mice.

## Introduction

Alzheimer’s disease (AD) is defined by accumulation of amyloid-β (Aβ) plaques and neurofibrillary tau tangles in the brain, leading to neurodegeneration and cognitive dysfunction^1^. The need for deeper molecular understanding underlying AD pathogenesis and the discovery of effective therapeutics has led to the development of transgenic mouse models mimicking Aβ and tau pathologies. These neuropathological alterations in the brain are directly visualized by dyes, providing valuable information as an indicator of disease progression. As plaques and tangles share a common β-sheet-rich structure, both on the brain tissue are stained by β-sheet-binding dyes such as thioflavin S (ThS)^2,3^. Besides these AD-related proteins, ThS also binds to other β-sheet-containing deposits such as Lewy bodies of α-synuclein^4,5^.

5XFAD is a well-validated and widely used transgenic mouse model of AD that possesses a total of five mutations in amyloid precursor protein (APP) and presenilin (PSEN1) genes, which are involved in Aβ production. Swedish (K670N/M671L), Florida (I716V), and London (V717I) mutations in APP genes, and M146L and L286V mutations in PSEN1 genes contribute to the rapid development of Aβ deposits and progressive cognitive decline in 5XFAD mice. As the 5XFAD mouse model was originally generated to only develop amyloid pathology, single-staining with ThS is widely used for Aβ plaque detection^6–11^.

In Aβ-expressing transgenic mouse models such as 5XFAD, the therapeutic effects of Aβ-targeting drugs can be evaluated through the changes in the amount of ThS-stained aggregates. However, while evaluating the efficacy of Aβ-targeting drugs in ThS-stained brains of 5XFAD mice, we found that drugs that were previously shown to effectively reduce plaques in APP/PS1 double transgenic mice had less effect in 5XFAD mice. Therefore, we hypothesized that 5XFAD mice express β-sheet-rich proteins other than Aβ, which may have ostensibly caused such results in ThS-stained mice brains. To identify the protein component of ThS-positive aggregates, male 5XFAD mice from 7 to 8 months of age and female from 5 to 6 months of age were prepared. Their brain sections were double-stained by ThS with anti-Aβ(1–16) monoclonal antibody 6E10, anti-Aβ(17–24) monoclonal antibody 4G8, anti-phosphorylated tau monoclonal antibody AT8, or anti-α-synuclein monoclonal antibody (Table 1). For further validation of our hypothesis that the non-specific detection of ThS against multiple protein aggregates may complicate Aβ quantification and Aβ-targeting drug evaluation in 5XFAD mice, an Aβ-dissociating chemical EPPS at a concentration of 100 mg/kg was orally administered to 6-month-old male mice in drinking water for six weeks (Table 1)^12^. Subsequently, the brains of vehicle- or EPPS-treated mice were double-stained by ThS with either 6E10 or AT8 to compare the differences between the number of ThS-positive aggregates and each antibody stained accumulates.

**Table 1:** Mice preparation for histochemical analyses and drug administration.

Age (months)	Gender	Number of mice	Markers	Brain regions
7–8	Male	3	ThS/6E10	Whole Cortex Hippocampus
			ThS/AT8	
			ThS/α-Synuclein	
5–6	Female	3	ThS/6E10	Whole Cortex Hippocampus
			ThS/AT8	
			ThS/α-Synuclein	
5–6	Female	3	ThS/4G8	Whole Cortex Hippocampus
7.5 (including 6 weeks of drug administration)	Male	5 (Vehicle)	ThS/6E10	Whole Cortex Hippocampus
		4 (EPPS)	ThS/AT8	

## Results

### ThS-positive and AT8-positive phospho-tau aggregates found in 5XFAD mouse brain

**Figure 1:**
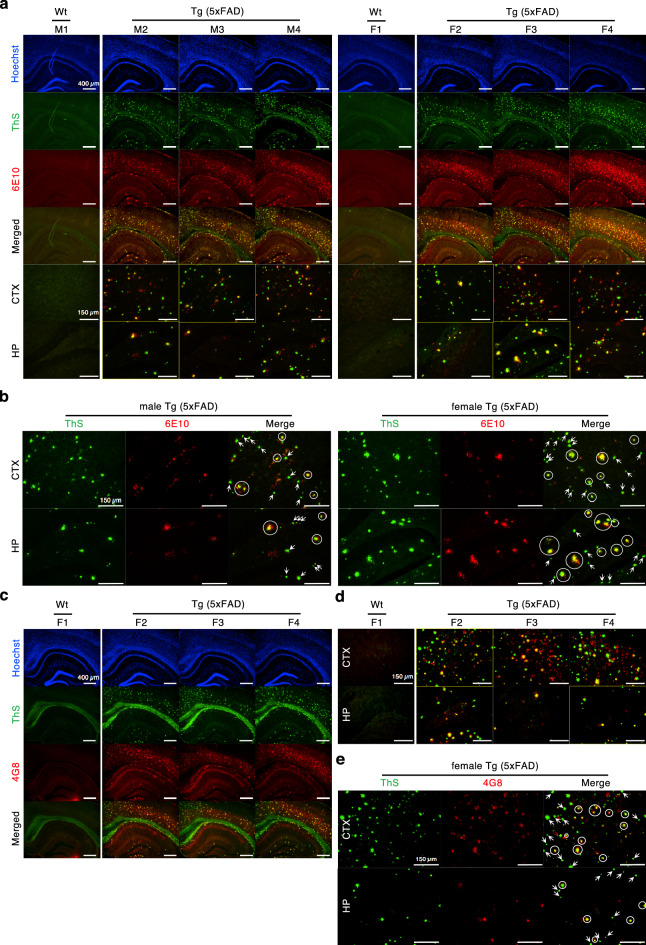
ThS stains Aβ plaques in 5XFAD mice brains and other protein aggregates as well. (**a**) The brains were double-stained by ThS and 6E10 anti-Aβ antibody. 7–8-month-old male (M2–M4) and 5–6-month-old female (F2–F4) 5XFAD mice were used. The brain of age-matched B6SJL wild type male (M1) or female mouse (F1) was also stained as a control. Hoechst 33342 was applied for nuclear counterstaining (scale bars = 400 µm, 150 µm). (**b**) The representative image of ThS- or 6E10-stained brains, which is indicated as yellow boxed images in (**a**) (scale bars = 150 µm). In merged images, yellow dots in white circles represent the double-staining of ThS and 6E10, whereas green dots marked by white arrows indicated 6E10-negative, ThS-positive aggregates. (**c**) ThS and 4G8 double-stained female 5XFAD brains at age of 5–6-month-old (F2–F4). The brain of age-matched B6SJL wild type female mouse (F1) was also stained as a control. Hoechst 33342 was used for nuclear counterstaining (scale bars = 400 µm). (**d**) Merged images of ThS and 4G8 double-staining (scale bars = 150 µm). (**e**) The representative image of ThS- or 4G8-stained brains, which is indicated as yellow boxed images in (**d**). In merged images of cortical and hippocampal regions, yellow dots in white circles indicted the double-staining of ThS and 4G8, while green dots marked by white arrows represented 4G8-negative, ThS-positive aggregates (scale bars = 150 µm). wt, wild type; Tg, transgenic; ThS, thioflavin S; CTX, cortex; HP, hippocampus.

To compare the type and the quantity of protein aggregates stained by ThS and aforementioned antibodies in the brain of 5XFAD, male (n = 3) and female (n = 3) 5XFAD mice were sacrificed at 7–8 months and 5–6 months of age, respectively. As female mice develop cerebral Aβ burden faster than males^8^, we used females one month younger than males in this study. For Aβ plaque detection, mouse brain sections were double-stained with ThS and 6E10, which is reactive to residue 1–16 of Aβ (Fig. 1a). In 5XFAD mouse brain, β-sheet-rich protein aggregates detected by ThS were thought to be Aβ plaques, which can be also stained by 6E10. However, besides the co-stained Aβ plaques (white circles in Fig. 1b), there was a significant number of 6E10-negative aggregates that were positive for ThS, in cortical and hippocampal regions of both male and female brains (white arrows in Fig. 1b). In both male and female mouse brains, the number of 6E10-negative aggregates, which appeared as green dots by only ThS fluorescent signal, was more than that of Aβ plaques shown as yellow dots by merged fluorescence (Fig. 1b). This observation seems improbable because 6E10 detect not only dense core plaques but also diffuse plaques, while ThS can only label dense core plaques in the brain of APP/PSEN1-expressing mouse models^13,14^. In addition, we confirmed the possibility that truncated Aβ species appears to be non-Aβ, represented as green fluorescent signals, because 6E10 binds to N-terminus of Aβ. The brain of female 5XFAD mice (n = 3) at 5–6 months of age were double-stained with ThS and 4G8, which is reactive to residue 17–24 of Aβ (Fig. 1c–e). As a similar result of 6E10 staining, along with the numerous Aβ plaques double-stained by ThS and 4G8 (white circles in Fig. 1e), 4G8-negative aggregates were also observed (white arrows in Fig. 1e) in both cortical and hippocampal regions.

**Figure 2:**
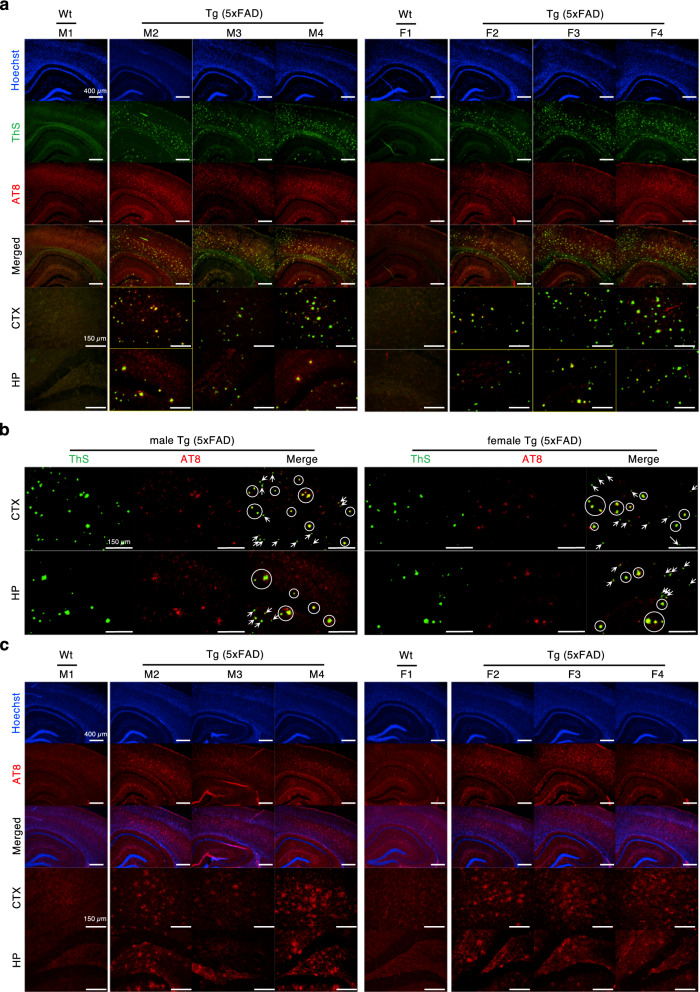
P-tau aggregates in 5XFAD mice brains were stained by AT8 antibody and ThS. (**a**) The brains were double-stained by Thioflavin S and AT8 anti-phosphorylated tau antibody. We used male (M2-M4) and female (F2-F4) 5XFAD mice at 7–8 months and 5–6 months of age, respectively. The brain of age- and gender-matched B6SJL wild type male (M1 and F1) was also stained as a control. Hoechst 33342 was used for visualization of nuclei (Scale bars = 400 µm, 150 µm). (**b**) The representative image of ThS- or AT8-stained brains, which is indicated as yellow boxed images in (**a**) (scale bars = 150 µm). Yellow dots of overlapped fluorescence in white circles represent the aggregated p-tau double-stained by ThS and AT8, whereas green dots marked by white arrows indicate ThS-positive aggregates that were not p-tau aggregates. (**c**) Single-labeling with AT8 antibody showed 5XFAD mouse brains exhibit significant amount of aggregated p-tau compared to wild type mouse brains (Scale bars = 400 µm, 150 µm). wt, wild type; Tg, transgenic; ThS, thioflavin S; CTX, cortex; HP, hippocampus.

**Figure 3:**
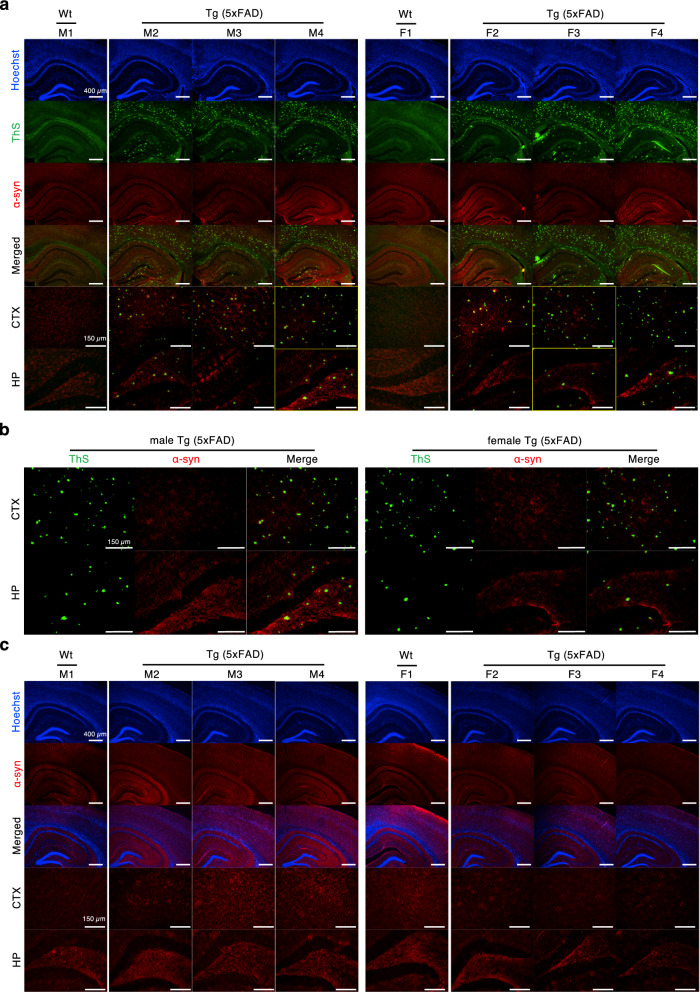
α-Synuclein aggregates were not detected in 5XFAD mice brains. (**a**) The brains were double-stained by Thioflavin S and AT8 anti-α-synuclein antibody. We used male (M2–M4) and female (F2–F4) 5XFAD mice at 7–8 months and 5–6 months of age, respectively. Age- and gender-matched B6SJL wild type male (M1 and F1) was used for a control (scale bars = 400 µm, 150 µm). (**b**) The representative image of ThS- or α-synuclein-stained brains, which is indicated as yellow boxed images in (**a**). Several green dots of ThS fluorescence were observed but no overlapped signal was detected in double-staining by ThS and α-synuclein (scale bars = 150 µm). (**c**) Single-labeling with α-synuclein antibody displayed no remarkable α-synuclein aggregates in 5XFAD mouse brains compared to wild type mouse brains (scale bars = 400 µm, 150 µm). wt, wild type; Tg, transgenic; ThS, thioflavin S; α-syn, α-synuclein; CTX, cortex; HP, hippocampus.

To identify the components of ThS-positive protein aggregates that were not stained by 6E10 and 4G8, brain sections were double-stained with ThS and AT8 (Fig. 2a) or α-synuclein antibody (Fig. 3a). AT8 staining revealed aggregated tau composed of phosphorylated tau at both Ser202 and Thr205 in brains of both genders, and the labeled structures overlapped with ThS (white circles in Fig. 2b). In brains stained with α-synuclein antibody, which recognizes amino acids 15–123 of α-synuclein, both genders of 5XFAD mice and wild type mice did not display detectable signals (Fig. 3a,b).

To confirm that yellow fluorescent signals in merged images were not due to the spectral overlap of ThS, we performed single-labeling with AT8 (Fig. 2c) or α-synuclein antibody (Fig. 3c). While AT8 single-staining showed a significant amount of tau aggregates in male and female 5XFAD mouse brains compared to age- and gender-matched wild type mouse brains, single-staining with α-synuclein antibody did not demonstrate visible fluorescence of α-synuclein. Therefore, we concluded that both male and female 5XFAD mice express aggregated phospho-tau (p-tau) that were detected by ThS.

### Complicated interpretation of Aβ-targeting drug upon ThS brain staining

**Figure 4:**
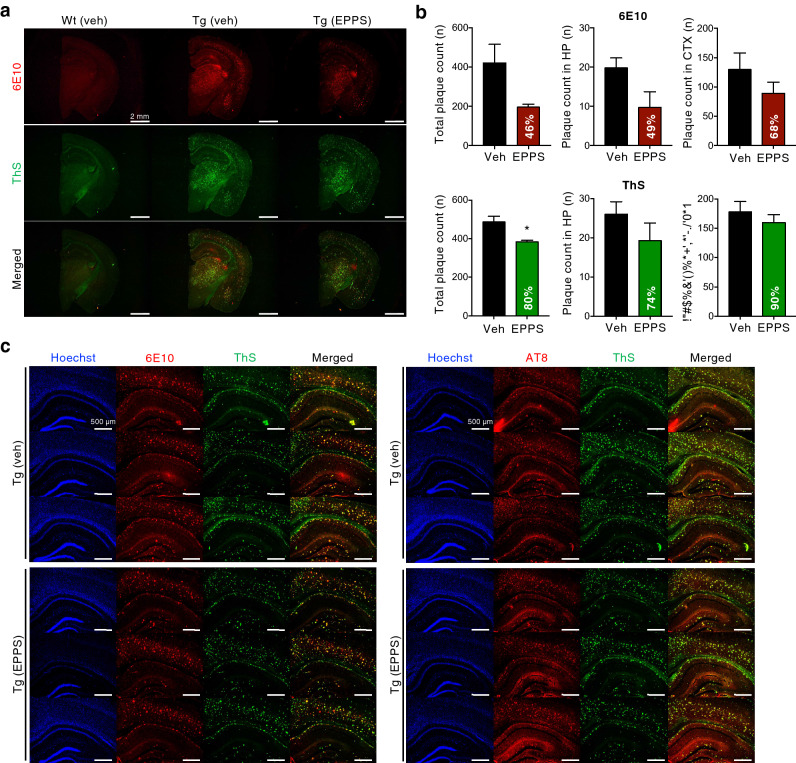
The different number of plaques were detected in 6E10- and ThS-stained 5XFAD mouse brains after the treatment of Aβ-disaggregating drug, EPPS. (**a**) The representative 6E10- and ThS-stained fluorescence half-brain images of the vehicle-treated wild type mouse, and vehicle- and EPPS-treated 5XFAD mice (scale bars = 2 mm). (**b**) Quantification of Aβ plaques in whole, hippocampal, and cortical regions of 6E10- or ThS-stained half-brains. The amount of remaining aggregates of EPPS group shown as a percentage in the bar graphs were normalized to the plaque numbers of vehicle group (100%). Significance was tested by unpaired two-tailed student’s *t*-test (*P < 0.05, other comparisons were not significant). (**c**) Individual and merged brain images of the vehicle- and EPPS administered- groups stained with ThS and 6E10 or AT8 antibody (scale bars = 500 µm). wt, wild type; Tg, transgenic; veh, vehicle; ThS, thioflavin S; CTX, cortex; HP, hippocampus.

To show the discrepancies between ThS and 6E10 staining in Aβ detection, 6-month-old male 5XFAD mice were administered 100 mg/kg of EPPS, a previously reported Aβ-plaque-dissociating molecule^12,15^, via drinking water for six weeks. After the administration, the disaggregating effect of EPPS on Aβ plaques in the brain was evaluated by histochemistry using ThS with 6E10 or AT8 (Fig. 4a-c). Congruous with the previous study^12,15^, a greater reduction in the number of Aβ plaques was observed in brains stained with 6E10 (Fig. 4b); 46% (total area), 49% (hippocampus), and 68% (cortex) of 6E10-stained plaques remained after EPPS treatment when plaque numbers of the vehicle group were normalized to 100%. However, ThS staining diminished the difference of aggregate amount between the two groups; 80% (total area), 74% (hippocampus), and 90% (cortex) of aggregates in the brain of EPPS-administered mice compared to vehicle-administered mice. The average percentages of plaque reduction by EPPS administration in 6E10 and ThS staining were 45.7% and 18.7%, respectively (Supplementary Fig. S1). Collectively, we deduced that this discrepancy between two staining methods may be attributed to that ThS-positive p-tau aggregates complicate the quantification of plaques in the brains of 5XFAD mice, leading to incorrect validation of drug efficacy (Fig. 4a–c). In conclusion, cross-checking data with an Aβ-specific antibody is recommended in the evaluation of Aβ-targeting drugs.

## Discussion

Here, we report that single-staining with ThS for Aβ plaque measurements in the 5XFAD mouse brains can lead to incorrect interpretation because 5XFAD exhibits ThS-positive p-tau aggregates in their brains. We observed ThS-labeled insoluble protein aggregates that were not positive for anti-Aβ antibodies, 6E10 or 4G8, in the cortical and hippocampal regions of both male and female 5XFAD mice. Considering the possibility that the ThS + /anti-Aβ aggregates– could be composed of other aggregation-prone proteins, the brain sections of 5XFAD mice were stained with antibodies against phosphorylated tau and α-synuclein. Through double-staining with ThS and AT8, we deduced that the ThS + /anti-Aβ aggregates—were p-tau aggregates, ThS + /anti-hyperphosphorylated tau + . To test whether Aβ quantification with ThS in 5XFAD mouse brains interferes the evaluation of the efficacy of anti-Aβ drug, 5XFAD mice were orally administered an Aβ-dissociating agent, EPPS, and their brains were double-stained by ThS with 6E10 or AT8. We found that ThS-stained brains displayed a smaller extent of EPPS-induced plaque reduction than in the same brains stained with 6E10, demonstrating the need for extra attention in the use of ThS as an Aβ-detecting probe in 5XFAD mice.

As the 5XFAD mouse model was originally designed for the investigation of APP- and Aβ-related pathology, this model is not expected to develop significant tauopathy in the brain. Subsequently, tau aggregates were reported to be absent in this model when using a phosphorylated tau antibody recognizing phosphorylation at Ser199/202^8,16^. However, recent studies revealed that 5XFAD mice display a gradual increase of total tau level in cerebrospinal fluid^17^ as well as significant aggregated p-tau stained by AT8 recognizing phosphorylation at Ser202 and Thr205^18,19^. Furthermore, accumulating reports indicate that a pretangle state, in which tau is phosphorylated and accumulated^20^, was observed in Aβ-overexpressing transgenic mouse models including 5XFAD mice^21–25^. Consistent with these results, we observed ThS-positive tau phenotypes in the brains of both male and female aged 5XFAD mice by double-staining with ThS and AT8. As the tau hyperphosphorylation plays a critical role as a trigger for the development of neurofibrillary tangles, further investigation is needed to demonstrate the exact mechanisms on the alterations of endogenous tau in pretangle state of Aβ-overexpressing mouse models. The further analyses are also required to investigate the phosphorylation of the other pathologic tau epitopes in the brain of 5XFAD mice, except for Ser202 and Thr205 that were detected by AT8 antibody. Additionally, an in-depth study on whether these p-tau aggregates were neurofibrillary tau tangles or phospho-tau within dystrophic neurites surrounding Aβ plaques is also warranted.

To conclude, due to the presence of p-tau aggregates in the brain of 5XFAD mice, the use of ThS in Aβ quantification in this model requires caution since binding property of ThS against both Aβ and tau aggregates can lead inaccurate results. As these findings were only observed in the specific age of mice, further studies are needed to confirm age-dependent accumulation of tau in the brain of both male and female 5XFAD mice. Furthermore, the presence of phosphorylated tau at other epitopes also needs to be investigated.

## Materials and methods

### Animals

For histochemical analysis, male 5XFAD mice (strain name; B6SJL-Tg(APPSwFlLon,PSEN1*M146L*L286V) 6799Vas/Mmjax) from 7 to 8 months of age (n = 3) and female from 5 to 6 months of age (n = 6) were obtained from Jackson Laboratory (USA). Age- and gender-matched wild type mice (n = 1, each) were used as controls. All mice were bred and provided constant temperature, humidity, and 12:12 h light–dark cycle in the animal facility of Yonsei University. Mice were given ad libitum access to water and food. All animal experiments were carried out in accordance with the National Institutes of Health guide for the care and use of laboratory animals (NIH Publications) as well as the ARRIVE guidelines and approved by the Animal Institutional Animal Care and Use Committee of Yonsei University (Korea, IACUC-202003–1038-01).

### Drug administration

A dose of 100 mg/kg of EPPS (Sigma-Aldrich, USA), a previously reported Aβ-dissociating drug candidate, was freely administered to 6-month-old male 5XFAD mice (n = 4) via drinking water for six weeks. After the administration, all mice were deeply anesthetized by intraperitoneal injection of 4% avertin (2,2,2-Tribromoethanol, Sigma-Aldrich, USA) and sacrificed by cervical dislocation to obtain brain tissue samples and compare the number of labeled plaques by 6E10 antibody and ThS. Age- and gender-matched 5XFAD transgenic mice (n = 5) and B6/SJL wild type mice (n = 4) were also sacrificed as controls.

### Histochemistry

All mice were deeply anesthetized by intraperitoneal injection of 4% avertin for the brain extraction. After perfusion with 0.9% NaCl, mice were sacrificed by cervical dislocation and extracted brains were fixed in 4% paraformaldehyde (Biosesang, Korea). After 24 h of fixation, brains were immersed in 30% sucrose for 2 days. For immunofluorescence staining, 35 µm-thick frozen sections were incubated with 6E10 (Biolegend, USA, Catalog# SIG-39320, 1:200), 4G8 (Biolegend, USA, Catalog# SIG-39220, 1:200), AT8 (Invitrogen, USA, Catalog# MN1020, 1:200), or α-synuclein (BD Transduction Laboratories, USA, Catalog# 610786, 1:250) antibody diluted in 5% horse serum (Gibco, USA), then with Alexa555-conjugated secondary antibody (1:200 in PBS). Each stained section was incubated in 500 µM of Thioflavin S (ThS, Sigma-Aldrich, USA) dissolved in 50% ethanol for 7 min for ThS double-staining. Hoechst 33342 (10 µg/mL, Sigma-Aldrich, USA) was used to observe nuclear morphology. All images were taken using a fluorescence microscope (Leica DM2500, Germany). The number and area of plaques detected by ThS and 6E10 were quantified using Image J software.

### Statistical analysis

Graphs were obtained by GraphPad Prism 7 and statistical analyses were performed using unpaired two-tailed Student’s *t*-test. All data were represented as mean ± SEM.

## Supplementary information

Supplementary Information.
