# Supplementary material for: Thioflavin-positive tau aggregates complicating quantification of amyloid plaques in the brain of 5XFAD transgenic mouse model
Source: Sci Rep. 2021 Jan 15;11:1617. doi: 10.1038/s41598-021-81304-6 (PMC7810901; doi:10.1038/s41598-021-81304-6)
Supplement: Supplementary file 1 — Supplementary Information. [file 41598_2021_81304_MOESM1_ESM.pdf]

Thioflavin-positive tau aggregates complicating quantification of amyloid plaques in the brain of 5XFAD transgenic mouse model

Jisu Shin[1], Sohui Park[1], HeeYang Lee[1], and YoungSoo Kim[1,*]

[1]Department of Pharmacy, Yonsei University, Incheon 21983, Republic of Korea

[*]Correspondence and requests for materials should be addressed to Y.K. (y.kim@yonsei.ac.kr +82-32-749-4523)

Supplementary Figure

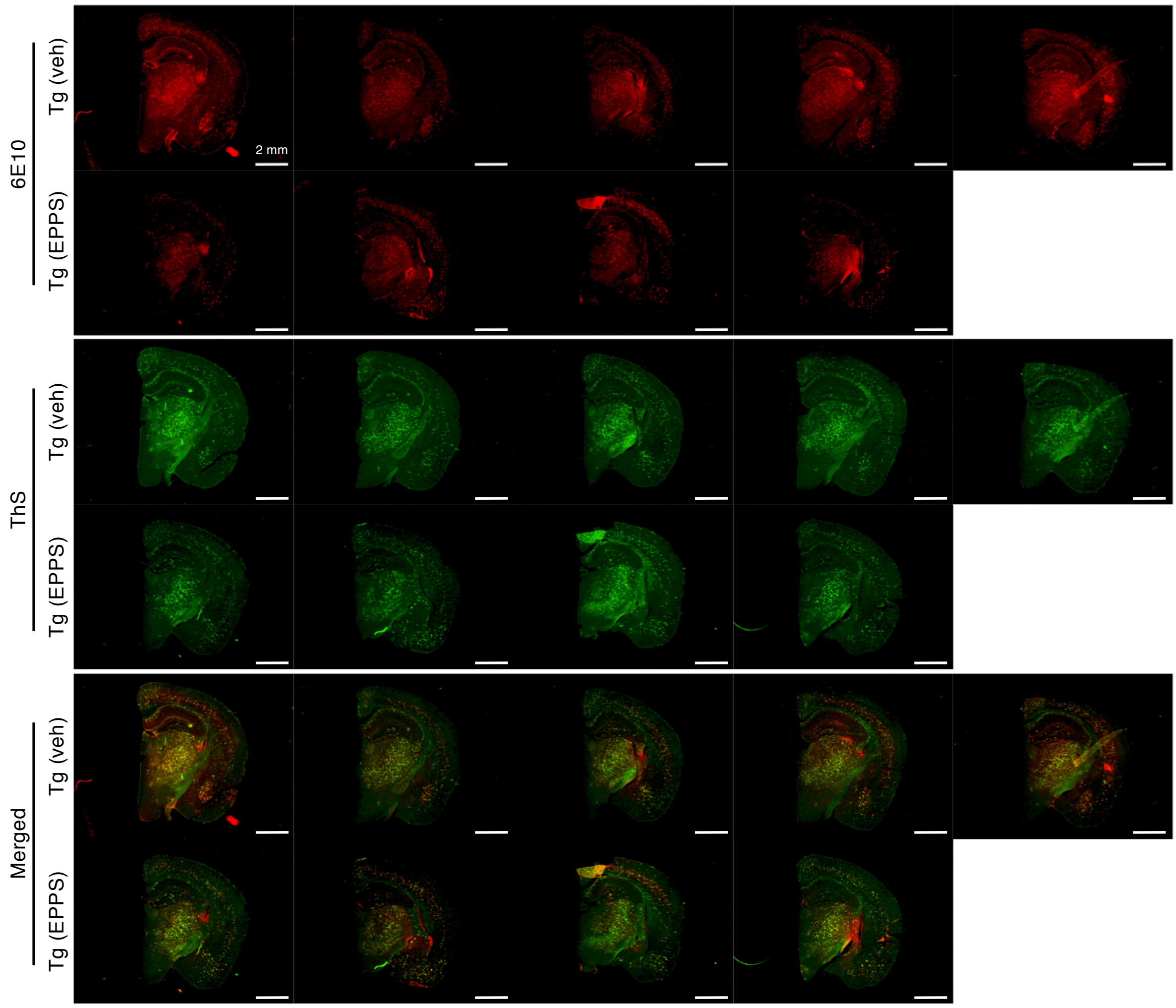

Supplementary Figure S1. The brain images of all mice treated vehicle or EPPS. All images of 6E10- and ThS-stained brain sections after the administration of vehicle or Aβ-disaggregation drug, EPPS, to 6-month-old male 5XFAD mice (Scale bars = 2 mm). Abbreviations: Wt, wild type; Tg, transgenic; veh, vehicle; ThS, thioflavin S.
